# Neuroprotective Effects of Mushroom Biomass Digestive Fractions and Gut Microbiota Metabolites in Microglial and *Caenorhabditis elegans* Models of Neurodegeneration

**DOI:** 10.3390/nu17243867

**Published:** 2025-12-11

**Authors:** Helena Araújo-Rodrigues, Lidia Garzón-García, Ana Sofia Salsinha, João Bettencourt Relvas, Freni K. Tavaria, Celestino Santos-Buelga, Ana M. González-Paramás, Manuela E. Pintado

**Affiliations:** 1 Universidade Católica Portuguesa, CBQF—Centro de Biotecnologia e Química Fina—Laboratório Associado, Escola Superior de Biotecnologia, Rua Diogo Botelho 1327, 4169-005 Porto, Portugal; hrodrigues@ucp.pt (H.A.-R.); asalsinha@ucp.pt (A.S.S.); ftavaria@ucp.pt (F.K.T.); 2 Glial Cell Biology, i3S-Instituto de Investigação e Inovação em Saúde, Universidade do Porto (UP), 4200-135 Porto, Portugal; jrelvas@ibmc.up.pt; 3 Grupo de Investigación en Polifenoles (GIP-USAL), Campus Miguel de Unamuno, Universidad de Salamanca, 37007 Salamanca, Spain; lidiagarzon@usal.es (L.G.-G.); csb@usal.es (C.S.-B.); paramas@usal.es (A.M.G.-P.); 4 IBMC—Instituto de Biologia Molecular e Celular, Universidade do Porto (UP), 4200-135 Porto, Portugal

**Keywords:** mushroom biomass, neuroprotective effects, human microglial cell line (HMC3), ROS production, *Caenorhabditis elegans*, hyperphosphorylated Tau and amyloid toxicity, chemotaxis and paralysis experiment

## Abstract

**Background:** The accumulation of β-amyloid plaques, neurofibrillary tangles, and neuroinflammation are key hallmarks of Alzheimer’s disease (AD). Reactive oxygen species (ROS) act as major triggers and amplifiers of neuroinflammatory responses, contributing to immune dysregulation and neuronal damage. Despite extensive research, no effective therapy halts or reverses AD progression, emphasizing the need for alternative preventive strategies, including the use of natural compounds. **Objectives:** This study evaluated the neuroprotective effects of simulated digestive fractions (permeate fraction) of mushroom biomass (MB)—*Trametes versicolor* (TV), *Hericium erinaceus* (HE), and *Pleurotus ostreatus* (PO)—and key gut microbiota-derived metabolites, such as short-chain fatty acids (SCFAs) and γ-aminobutyric acid (GABA) on ROS production in human microglial cells (HMC3) and in transgenic *Caenorhabditis elegans* models exhibiting hyperphosphorylated Tau and β-amyloid-induced toxicity. **Methods:** Cell viability and ROS production were assessed in HMC3 cells treated with mushroom fractions and metabolites. Chemotaxis and paralysis assays were performed in transgenic *C. elegans* strains expressing hyperphosphorylated Tau or β-amyloid proteins. **Results:** Mushroom digestive fractions and SCFAs significantly decreased ROS levels in HMC3 cells. Moreover, mushroom digestive fractions, butyric acid, and GABA improved behavioral outcomes in *C. elegans*, enhancing chemotaxis and delaying paralysis. These effects were dose-dependent and varied among mushroom species and metabolites. **Conclusions:** Mushroom-derived digestive fractions and microbiota-related metabolites exhibit neuroprotective activity by modulating oxidative stress and mitigating neurodegeneration-associated behaviors. Diets enriched with such MBs may support preventive strategies for neurodegenerative diseases. Further research is required to elucidate the molecular mechanisms underlying these protective effects and their translational potential for human neurodegenerative diseases.

## 1. Introduction

Alzheimer’s disease (AD) is a progressive neurodegenerative disorder characterized by memory loss and cognitive decline. Its causes are not fully understood, but the accumulation of β-amyloid plaques and neurofibrillary tangles is a well-recognized hallmark [1]. β-amyloid (Aβ) peptides, consisting of 38 to 42 amino acids, are a cleavage product of the transmembrane amyloid precursor protein (APP). Under normal conditions, APP is hydrolyzed via a non-amyloidogenic pathway involving sequential α-secretase and γ-secretase complexes’ action, generating a soluble *N*-terminal peptide. However, when APP is sequentially hydrolyzed by β- and γ-secretases, a *C*-terminal membrane-bound fragment is released, generating Aβ peptides (Aβ_1–40_ and Aβ_1–42_). These peptides aggregate to form extracellular plaques, disrupting neurotransmission and synaptic function [1,2].

Tau protein, a microtubule-associated protein that interacts with tubulin to promote assembly and maintain stability, is essential for proper neuronal function and axonal transport. Under pathological conditions, the abnormal hyperphosphorylation of this protein results in microtubule depolymerization and conformational alteration, promoting aggregation of this microtubule-associated protein. This disrupts its binding to microtubules, leading to the formation of neurofibrillary tangles that negatively affect neuronal structure and function [1,2].

Another critical event in AD progression is microglial overactivation, which triggers the expression of inflammatory mediators, leading to neuroinflammation and neuronal damage [3]. Along with neuroinflammation, oxidative stress is a hallmark of neurodegeneration, contributing to disease progression. Indeed, protein aggregation and neuronal damage activate disease-associated microglia via damage-associated molecular patterns. This ultimately results in persistent inflammation and the generation of reactive oxygen species (ROS). Thus, although it is unlikely that ROS are the primary triggers of inflammation in AD, they may act as secondary messengers in microglia to propagate the inflammatory state, leading to immune dysregulation [2,4]. Taken together, these mechanisms highlight the complexity of AD pathology and suggest that factors beyond the central nervous system may also influence disease progression.

In this context, growing evidence indicates that the gut microbiota contributes to the pathogenesis and progression of neurodegenerative diseases, including AD [5,6,7,8]. The gut microbiota metabolizes dietary polysaccharides and other compounds with prebiotic potential, producing key metabolites, including short-chain fatty acids (SCFAs; acetic, propionic, and butyric acids), amino acids (e.g., γ-aminobutyric acid—GABA), and vitamins (e.g., vitamin K and B). Alterations in microbiota composition and function have been associated with AD, and reduced SCFAs and GABA levels have been reported in patients. These microbial metabolites play an essential role in host homeostasis and can influence neurological health through bidirectional gut-brain communication [5,6,7,8]. Therefore, dietary strategies capable of modulating microbiota composition emerge as promising approaches to support brain health. Despite significant research efforts, no effective treatment is currently available to reverse AD progression, highlighting the need for alternative strategies, particularly the exploration of natural compounds with neuroprotective potential and gut microbiota-modulating potential [8].

Mushrooms are rich in bioactive compounds, including polysaccharides (α-glucans, β-glucans, and chitin), proteins (lectins), lipids (oleic and linoleic acids), vitamins, minerals, and terpenes. Beyond their nutritional profile, many species exhibit prebiotic, immunomodulatory, anti-inflammatory, antioxidant, and neuroprotective effects [9,10,11]. Notably, *Trametes versicolor* (TV) and *Hericium erinaceus* (HE) have been reported to improve hippocampal function, activate defense mechanisms relevant to AD, and mitigate symptoms in Parkinson’s disease (PD) models [12,13,14,15,16,17,18]. Importantly, mushrooms may exert their health benefits not only through direct absorption of bioactive metabolites after digestion but also by modulating the gut microbiota [10]. Thus, mushroom-derived prebiotic effects, combined with their rich bioactive composition, emerge as a promising therapeutic strategy for further exploration [19,20]. Despite the promising effects of mushroom-based diets, the mechanisms responsible for these benefits are still poorly understood. These effects may involve direct absorption of bioactive compounds after digestion, effects of microbial metabolites, or a synergistic interaction between both pathways. Clarifying these mechanisms is crucial for supporting the development of targeted dietary interventions for neurodegenerative diseases.

To investigate these mechanisms, relevant *in vitro* and *in vivo* models are required. Human microglial cell lines, constituting approximately 5–10% of brain cells, are widely used to study neurodegenerative processes, particularly oxidative stress and inflammation. Microglia can initiate neuroinflammatory responses upon exposure to external stimuli, including ROS production [21,22]. Complementarily, *Caenorhabditis elegans* is a well-established model organism due to its high homology and conservation with human metabolic and disease pathways. This free-living soil nematode has a short life cycle and is easy to maintain and handle [23,24,25,26]. Its well-defined genome and availability of transgenic strains make it an ideal model for studying neuronal development, function, and behavior. This organism has been widely used to explore the effects of bioactive dietary compounds on brain health, particularly in AD models [23,24,25], where suppression of Aβ peptide and Tau protein aggregation has shown therapeutic promise. Numerous transgenic strains expressing human pathological proteins, such as abnormal Aβ and tauopathy models, are used to evaluate the anti-AD effects of food extracts [27,28].

The main aim of this work was to evaluate the neuroprotective effects of mushroom biomass (MB). The selected MB species include TV and HE, both recognized for their medicinal properties and reported neuroprotective and anti-inflammatory effects [12,13,17,29], as well as *Pleurotus ostreatus* (PO), a widely consumed edible mushroom with reported prebiotic potential [30,31]. This study assessed simulated digestive fractions (representing potentially directly absorbed metabolites) and gut microbiota-derived metabolites resulting from gut microbiota metabolism (via human fecal fermentation) from these MB for their capacity to modulate ROS production in human microglial cells (HMC3) and to counteract Tau- and Aβ-induced toxicity in transgenic *C. elegans* models of AD. By integrating *in vitro* and *in vivo* approaches, this work provides a comprehensive evaluation of the neuroprotective potential of mushroom-derived macromolecules and gut microbiota metabolites, analyzing their influence on ROS production in the HMC3 cell line and on chemotactic behavior and paralysis in transgenic *C. elegans* models of AD.

## 2. Materials and Methods

### 2.1. Chemicals

α-amylase from *Bacillus licheniformis* (A4551; 70.94 U/mg), pancreatin from porcine pancreas (P7545; 8 U/mg), and bile salts (bile bovine B3883; 2.01 mmol/g) were purchased from Sigma-Aldrich (St. Louis, MO, USA), while rabbit gastric extract (RGE 15; 517.3 U/mg) was obtained from Lipolytech (Marseille, France). VWR Chemicals (Radnor, PA, USA) supplied the 3.5 kDa cut-off cellulose membrane (Pre-wetted RC Tubbing, Spectra/Por^®^ 6 Dialysis Membrane; 734–0652; Spectrum Laboratories, Rancho Dominguez, CA, USA). Regarding SCFAs standards, acetic acid (20104.323) was supplied by VWR Chemicals, while propionic acid (P1386), butyric acid (B10350-0), and GABA were obtained from Sigma-Aldrich.

Cell culture reagents Dulbecco’s modified Eagle’s medium (DMEM) + GlutaMAX™-I, fetal bovine serum (FBS), penicillin, streptomycin, and PrestoBlue reagent were purchased from Thermo Fisher Scientific (Waltham, MA, USA). At the same time, DCFDA/H2DCFDA—Cellular ROS Assay Kit was obtained from Abcam (Cambridge, UK). Clear-bottom 96-well plates were also acquired from Thermo Fisher Scientific.

Components for nematode growth medium (NGM) and lysogeny broth (LB), including cholesterol, peptone, agar, yeast extract, tryptone, nystatin, and ampicillin sodium salt, were supplied by Sigma-Aldrich (Madrid, Spain). Potassium monohydrogen phosphate, potassium dihydrogen phosphate, magnesium sulfate, and sodium monohydrogen phosphate were obtained from Merck (Darmstadt, Germany), while sodium chloride and calcium chloride were supplied by Panreac (Barcelona, Spain). Petri plates (Ø 35 and 60 mm) were provided by Brand GMBH (Wertheim, Germany). Additional reagents for *C. elegans* synchronization, chemotaxis, and paralysis assays, such as sodium azide and benzaldehyde, were purchased from Sigma-Aldrich; dimethyl sulfoxide (DMSO) and sodium hydroxide from Panreac, and ethanol and isoamyl alcohol from Merck.

### 2.2. Mushroom Biomass (MB) Samples

The MB species used in this study—TV, HE, and PO—were kindly provided by Mycology Research Laboratories Ltd. (Luton, UK) and Aneid-Produtos Farmacêuticos Lda (Cascais, Portugal). These MB were produced under Food Safety Certification. The dried milled MBs (particle size between 250 and 500 µm) were stored in a desiccator protected from light until analysis.

### 2.3. Fractions Resultant from the Simulated Digestive Process

To simulate gastrointestinal tract digestion, the standardized static digestion model outlined in the INFOGEST 2.0 *in vitro* static digestion protocol was followed, as described by Brodkorb et al. [32]. Briefly, the oral phase was simulated by mixing MB with α-amylase (75 U/mL) and simulated salivary fluid (1:1, *w*/*w*) at 37 °C for 2 min on an orbital shaker (MaxQ 6000; Thermo Scientific, Waltham, MA, USA) at 200 rpm. The gastric phase was initiated by adding simulated gastric fluid (1:1, *v*/*v*), adjusting the pH to 3.0, and adding rabbit gastric extract (pepsin at 2000 U/mL and lipase at 60 U/mL). The mixture was incubated at 37 °C for 2 h under agitation at 130 rpm. For the intestinal phase, simulated intestinal fluid (1:1, *v*/*v*) and bile salts (10 mM) were added, the pH was adjusted to 7.0, and the mixture was incubated for 30 min at 37 °C under agitation at 130 rpm, before adding pancreatin (100 U/mL) for a final 2 h digestion at 45 rpm. Following intestinal digestion, dialysis was performed using a 3.5 kDa cut-off cellulose membrane to mimic the passage through the duodenum and jejunum. As a control, a blank experiment with distilled water instead of MB was conducted under identical gastrointestinal digestion and dialysis conditions. The resulting fractions—the permeate (<3.5 kDa) and retentate (>3.5 kDa)—were freeze-dried, representing potential serum-available metabolites that could be absorbed after gastrointestinal digestion and colon-available fraction that could be fermented by gut microbiota. In this study, only the permeate fraction (<3.5 kDa), representing potentially serum-available metabolites after gastrointestinal digestion, was used for the biological assays.

### 2.4. Human Microglia Clone 3—(HMC3) Cell Line Assays

#### 2.4.1. Human Microglia Clone 3 (HMC3) Cell Line Maintenance

The HMC3 cell line (ATCC CRL-3304) was derived from primary cultures of human embryonic microglial cells immortalized by transfection with a plasmid encoding SV40 large T antigen [33]. Cells were cultured in DMEM supplemented with GlutaMAX™-I, 10% FBS, and antibiotics (100 U/mL of penicillin and 100 μg/mL of streptomycin). Cultures were maintained at 37 °C in a humidified 5% CO_2_ atmosphere [34].

#### 2.4.2. Cell Viability Analysis

Cell viability, measured through metabolic activity inhibition, following treatment with different concentrations of simulated digestive fractions (permeate fractions) and gut microbiota metabolites, was evaluated using the PrestoBlue™ reagent, according to the manufacturer’s instructions. Briefly, cells were seeded (1 × 10^4^ cells/well) in clear-bottom 96-well plates and allowed to adhere for 24 h. Treatments included simulated digestive fractions (5, 2.5, 1.25, and 0.63 mg/mL) and gut microbiota metabolites (GABA: 3.13, 6.25, 12.5, 25, and 50 mM; SCFAs: 6.3, 12.5, 25, 50, and 100 µM), applied for 24 h. For negative controls, cells were treated with 10% DMSO. PrestoBlue reagent was diluted 1:10 *v*/*v* in fresh medium and incubated with the cells for 1 h in the dark. Fluorescence was measured using a multi-detection plate reader (excitation: 560 nm; emission: 590 nm). All assays were performed in quadruplicate, with three independent replicates. The cell viability was calculated in relation to the positive control (cells without treatment) and represented in %.

#### 2.4.3. ROS Production

Intracellular ROS production was assessed using a permeable fluorescent dye 2′,7′-dichlorodihydrofluorescein diacetate (DCFH-DA), following the methodology described by Martínez Leo et al. [35], with minor modifications. Cells were seeded (1.5 × 10^4^ cells/well) in a 96-well black clear-bottom plate and incubated for 24 h. Subsequently, cells were treated with different concentrations of simulated digestive fractions (0.31, 0.63, 1.25, and 2.5 mg/mL) and gut microbiota metabolites (GABA: 3.13, 6.25, 12.5, and 25 mM; SCFAs: 12.5, 25, and 50 µM) for 24 h. After treatment, cells were washed with 100 μL of 1× assay buffer and incubated with 100 μL DCFH-DA (10 μM) for 45 min at 37 °C (dark). ROS inhibition was assessed by using 100 μM *tert*-butyl hydroperoxide (TBHP), a known inducer of oxidative stress. Negative controls consisted of untreated cells (basal ROS production), while cells exposed to TBHP without treatment served as positive controls (maximum ROS production). After DCFH-DA incubation, the solution was removed, and cells were treated with 100 μL of TBHP (100 μM) prepared in 1x assay buffer for 4 h to induce oxidative stress. Fluorescence was measured in a multi-detection plate reader at excitation and emission wavelengths of 485 and 535 nm, respectively. Data were normalized to basal ROS production (negative control) and expressed as % inhibition relative to the positive control. All experiments were conducted in quadruplicate and repeated independently three times, with three independent replicates.

### 2.5. Caenorhabditis Elegans Assays

#### 2.5.1. Strains Selection

The mutant strains used in this study included CL2355 - dvIs50 [pCL45 (snb-1::Abeta 1-42::3′ UTR(long) + mtl-2::GFP] I; CL2122 - dvIs15 [(pPD30.38) unc-54(vector) + (pCL26) mtl-2::GFP]; BR5270 - byIs161 [rab-3p::F3(delta)K280 + myo-2p::mCherry]; BR5271 - byIs162 [rab-3p::F3(delta)K280 I277P I380P + myo-2p::mCherry]; and CL4176 - dvIs27 [myo-3p::A-Beta (1-42)::let-851 3′UTR) + rol-6(su1006)] X. *Escherichia coli* OP50 strain was used to feed the worms. These strains were obtained from the Caenorhabditis Genetics Center (University of Minnesota, Minneapolis, MN, USA). The strains CL2355, CL2122, BR5270, and BR5271 were used for the chemotaxis assays, while the CL4176 strain was used for paralysis assays [23,24,28].

#### 2.5.2. Preparation of Nematode Growth Medium (NGM) Petri Plates and Bacterial Food Sources

To prepare the NGM, 3 g of NaCl, 17 g of agar, and 2.5 g of peptone were mixed with 975 mL of water and sterilized at 121 °C for 15 min. After cooling to 55 °C, medium was supplemented with 1 mL of CaCl_2_ (1 M), 1 mL of MgSO_4_ (1 M), 1 mL of cholesterol (5 mg/mL ethanol), 25 mL of buffer KPO_4_ (pH 6, 1 M), 1 mL of sodium ampicillin (50 mg/mL) and 1.75 mL of nystatin (1%). SCFAs, GABA, and simulated digestive fractions were added to the NGM before plate seeding. The acetic, propionic, and butyric acids (final concentration of 25, 50, 75, 100, and 150 µM) were dissolved in DMSO, while GABA (25, 50, 75, 100, 150, 250, 500, 1000, 1500, and 3000 µM) and simulated digestive fractions (1.5, 2.5, 3.5, and 5 mg/mL) in water.

To prepare a bacterial food source, a single bacterial colony of *E. coli* OP50 was inoculated into a sterilized LB (pH 7) containing 5% *w*/*v* NaCl, 10% *w*/*v* tryptone, and 5% *w*/*v* yeast extract. The culture was incubated overnight at 37 °C. The liquid culture was stored at 4 °C and used to seed NGM plates.

#### 2.5.3. *C. elegans* Maintenance and Age-Synchronization

All *C. elegans* strains were age-synchronized. Gravid worms (in the adult phase, capable of egg-laying) were either transferred to new NGM plates or subjected to bleaching.

For the BR5270 and BR5271 strains, gravid hermaphrodites were treated with NaOH (5 M) and bleach (1:2), alternating 30 s of vortexing with 30 s of rest. During bleaching, worms were dissolved in the bleaching solution, while eggs were resistant and remained intact. The resultant suspension was centrifuged for 1 min at 9500× *g,* and the supernatant was discarded. The pellet was washed six times with M9 buffer (3% *w*/*v* KH_2_PO_4_, 6% *w*/*v* Na_2_HPO_4_, 5% *w*/*v* NaCl, and 1% *v*/*v* MgSO_4_ 1 M). These strains were routinely propagated at 20 °C on NGM seeded with *E. coli* OP50 as a food source.

Other strains (CL2355, CL2122, and CL4176) were synchronized by transferring gravid worms to new plates, allowing 24 h for egg-laying. Adult worms were then removed, and the plate was kept at 16 °C.

**Figure 1:**
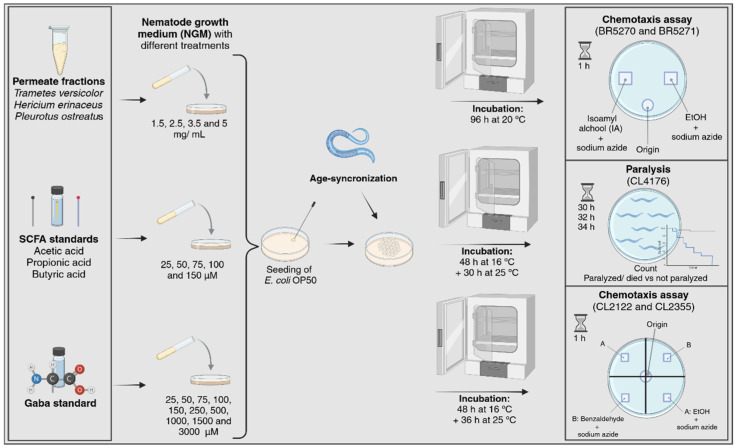
Scheme of the *C. elegans* experimental plan (plate preparation, age synchronization, chemotaxis, and paralysis assays). Created with www.Biorender.com.

The experimental plan outlined in this work is summarized in Figure 1, and the chemotaxis and paralysis assays are described in the following sub-sections. All *C. elegans* assays were performed by the same trained researcher using a standardized plate layout and fixed positions for each treatment, reducing subjectivity in scoring chemotaxis and paralysis phenotypes.

#### 2.5.4. Chemotaxis Assay with BR5270 and BR5271 Strains

The chemotaxis assay was performed as described by Fang et al. [36], with adjustments described by Garzón-García et al. [24]. Age-synchronized eggs of the BR5270 strain, expressing the pro-aggregant F3ΔK280 fragment of human Tau protein, were cultured on NGM plates seeded with *E. coli* OP50 at 20 °C for 96 h. Plates were prepared with treatment conditions and the corresponding controls (0.1% DMSO or 0.1% water). Control plates of the BR5270 strain were grown under the same conditions. At the 1-day-old adult phase, the worms were collected and washed three times with M9 buffer to remove residual *E. coli* OP50. Approximately 250 washed animals were transferred to the origin spot of each Ø 90 mm NGM plate. The plates were marked as illustrated in Figure 1. At two equidistant spots from the origin, 1 µL of sodium azide (1 M) was added to anesthetize the animals. Immediately, 1 µL of isoamyl alcohol (IA; 1% *w*/*w* in water) was placed at one spot (attractant site), while 1 µL of ethanol (EtOH; 99.5%) was placed in the opposite spot (neutral compound, control site). The plates were incubated for 1 h at room temperature. After the incubation period, the number of worms present at the attractant site, control site, and origin was recorded using a M205 FA microscope (Leica Microsystems, Wetzlar, Germany). All experiments were performed in triplicate. The chemotaxis behavior was expressed as the chemotaxis index (CI), calculated using the following formula:

$$CI=\frac{n[IA]-n[EtOH]}{n\left[IA\right]+n\left[EtOH\right]+n[Origin]}$$

#### 2.5.5. Chemotaxis Assay with CL2355 and CL2122 Strains

For the chemotaxis assay using transgenic *C. elegans* strains CL2355 and CL2122, the methodology described by Ayuda-Durán et al. [23] was followed, with minor alterations. Age-synchronized eggs of the CL2355 strain were grown on NGM plates seeded with *E. coli* OP50 with treatments (SCFAs, GABA, and simulated digestive fractions) and without treatments (controls containing 0.1% DMSO or 0.1% water). Its control strain, CL2122, was subjected to the same conditions. After 48 h at 16 °C, *C. elegans* plates were incubated at 25 °C for 36 h to induce Aβ expression in neuronal cells. The worms were collected and washed three times with M9 buffer to remove residual *E. coli* OP50. Approximately 100 washed animals were then transferred to the origin spot on each Ø 60 mm NGM plate (as illustrated in Figure 1). At each of the four spots on the plate equidistant from the origin, 1 µL of sodium azide (1 M) was added to immobilize the worms. In two opposite quadrants, 4 µL of ethanol (EtOH; 99.5%) was applied as a control, while in the other two opposite quadrants, 4 µL of benzaldehyde (B; 0.5% in ethanol) was used as the attractant. The plates were incubated for 1 h at room temperature. After incubation, the number of worms in the attractant and control quadrants was recorded under a M205 FA microscope. All experiments were conducted in triplicate. Chemotaxis behavior was expressed as the chemotaxis index (CI), calculated as:

$$CI=\frac{n[B]-n[EtOH]}{n\left[B\right]+n\left[EtOH\right]}$$

#### 2.5.6. Paralysis Assay

The paralysis assay was conducted following the method described by Ayuda-Durán et al. [23], with minor adjustments. Age-synchronized eggs of the *C. elegans* CL4176 strain were grown for 48 h at 16 °C in NGM plates seeded with *E. coli* OP50 (SCFAs, GABA, and simulated digestive fractions) and with the respective controls without treatments (controls containing 0.1% DMSO or 0.1% water). To induce Aβ expression in the muscle cells, the plates were transferred to a 25 °C incubator. After 30 h of incubation, approximately 100 worms were transferred to new NGM plates with the same treatments but without seeded *E. coli* OP50. The number of paralyzed (immobile or dead) and non-paralyzed animals, mechanically stimulated with a platinum wire pick, was scored under the microscope at 2 h intervals until 34 h. Each treatment was independently performed in triplicate.

### 2.6. Statistical Analysis

The statistics were performed using SPSS software (IBM SPSS Statistics; version 29.0; USA). The Shapiro-Wilk test (*n* < 50) was used to assess the normality of the data distribution. The homogeneity of variances across groups was evaluated using Levene’s test. A one-way analysis of variance (ANOVA) was conducted to determine statistically significant differences between groups, with a significance level of *p* < 0.05. For post-hoc multiple comparisons between groups, Tukey’s honest significant difference (HSD) test was applied. For the paralysis assay, *p*-values were determined by the log-rank test, with the same significance threshold (*p* < 0.05).

## 3. Results and Discussion

### 3.1. Mushroom Biomass (MB) Antioxidant Potential and Impact of Gastrointestinal Tract Simulation

As previously detailed by Araújo-Rodrigues et al. [37], the chemical characterization of MBs from TV, HE, and PO species revealed high contents of glucans (76.15 to 80.45% DW). The most prevalent glycosidic linkages detected in MBs were (1→4) linkages. Specifically, α-glucans and β-glucans with linkages different from (1→3)(1→6)-β-glucans accounted for 71.49% and 77.49%. The authors also found considerable amounts of proteins (4.08–6.28% DW), minerals (1.05–1.47% DW; e.g., phosphorus, magnesium, and potassium), and fatty acids (0.75–1.82% DW; e.g., palmitic, oleic, and linoleic acid) [37]. A subsequent study focusing on bioactive characterization and antioxidant potential of TV, HE, and PO MB (submitted to publication) indicated that these MBs are also a rich source of bioactive compounds, including antioxidants such as GABA, ergosterol, and ascorbic acid, as well as carotenoids, tocopherols, and free phenolic compounds, which contribute to their antioxidant capacity and protect against oxidative stress in a microglia cell line. Other studies have further corroborated the presence of significant levels of antioxidant compounds in mushrooms, supporting their role as free radical scavengers [38].

Concerning *in vitro* gastrointestinal tract simulation, recent data from the authors (not published yet) suggest that glucans were also the most abundant group in serum- and colon-available fractions. α-Glucans and β-glucans with linkages different from (1→3)(1→6)-β-glucans content in permeate fraction ranged from 38.80 to 40.08% DW. This quantification was validated by an intestinal permeability assay (Transwell^®^ membrane with a co-culture of Caco-2 and HT29). This fraction was also rich in low-molecular-weight peptides (<1.2 kDa), essential amino acids (Tyr, Val, Phe, and Leu), and phenolic compounds, exhibiting substantial antioxidant capacity. Regarding the retentate fraction (colon-available fraction), *in vitro* fecal fermentation assays demonstrated that MB modulates gut microbiota, enhancing the production of SCFAs and GABA, and increasing the relative abundance of beneficial bacterial genera such as *Bifidobacterium* and *Faecalibacterium*.

The present study focused on the serum-available, i.e., permeate fraction (<3.5 kDa), representing potential compounds that could be absorbed after gastrointestinal digestion. Given the inherent complexity of fecal fermentation samples, characterized by the presence of residual fecal inoculum and a diverse range of metabolic byproducts, analytical standards for the major metabolites were employed in this study to minimize potential analytical interferences. Nonetheless, it should be emphasized that the dialysis process provides a simplified estimation of absorption, which may not fully reflect the complexity of *in vivo* absorption, which is highly complex and influenced by several factors. Nevertheless, this approach provides a valuable first approximation for predicting potentially absorbable MB derived compounds. Besides, it is routinely used in functional food studies. Beyond permeate fractions derived from *in vitro* digestion of TV, HE, and PO MBs, the metabolites acetic acid, propionic acid, butyric acid, and GABA were also selected for further assessment regarding their effects on the HMC3 cell line and *C. elegans* models.

### 3.2. Microglia Cell-Based Assays with HMC3 Cells

#### 3.2.1. Cell Viability

**Figure 2:**
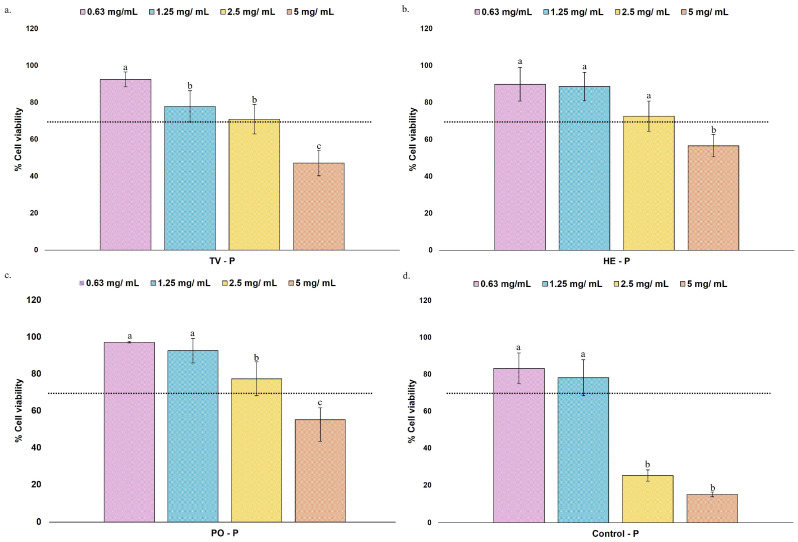
Cell viability (%) of HMC3 microglia cells after 24 h exposure to the simulated digestive fractions of TV (**a**), HE (**b**), PO (**c**) MBs, and gastrointestinal control (**d**) at different concentrations (0.63, 1.25, 2.5, and 5 mg/mL). Bars represent cell viability (mean ± SD), measured by the PrestoBlue assay in percentage (%) after 24 h of treatment relative to the positive control (HMC3 cells without treatment). Dotted lines represent the 70% cell viability threshold defined by ISO 10993-5, above which samples are considered non-cytotoxic and biologically relevant. A 10% DMSO solution was used as a negative control (0% viability). In each graph, bars with different superscript letters indicate statistical differences, assessed by one-way ANOVA followed by Tukey’s post hoc test (*p* < 0.05).

**Figure 3:**
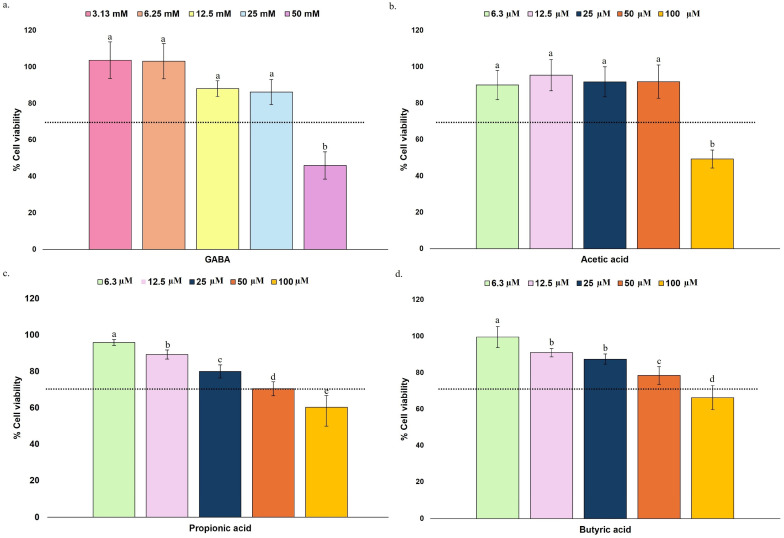
Cell viability (%) of HMC3 microglia cells after 24 h exposure to GABA (**a**), acetic acid (**b**), propionic acid (**c**), and butyric acid (**d**) gut microbiota metabolites at different concentrations (GABA: 3.13, 6.25, 12.5, 25, 50 mM; Acetic, propionic, and butyric: 6.3, 12.5, 25, 50, and 100 µM). Bars represent cell viability (mean ± SD), measured by the PrestoBlue assay in percentage (%) after 24 h of treatment relative to the positive control (HMC3 cells without treatment). Dotted lines represent the 70% cell viability threshold defined by ISO 10993-5, above which samples are considered non-cytotoxic and biologically relevant. A 10% DMSO solution was used as a negative control (0% viability). In each graph, bars with different superscript letters indicate statistical differences, assessed by one-way ANOVA followed by Tukey’s post hoc test (*p* < 0.05).

Cell culture models are widely recognized as relevant biological systems to assess the effects of food-derived compounds. Initial cell viability assays are essential to validate that observed biological effects are physiologically relevant and to ensure that observations are not due to cellular metabolic inhibition. The HMC3 cell line has been widely used as an *in vitro* model of human microglia to study neuroinflammatory and neurodegenerative processes [39,40]. In this study, HMC3 viability was evaluated after 24 h of exposure to simulated digestive fractions of MBs (permeate fractions), as well as gut microbiota metabolites, using the PrestoBlue metabolic assay (Figure 2 and Figure 3). Data were expressed as percentages relative to the positive control (HMC3 cells without treatment, set as 100% viability), whereas a 10% DMSO solution was used as a negative control to confirm complete metabolic inhibition (0% viability). The ISO 10993-5 threshold was followed, with viability values above 70% as non-cytotoxic and biologically relevant [41].

As far as the authors know, no studies have tested simulated digestive fractions, so the starting concentrations were based on previous reports where mushroom extracts were tested. The concentrations used for simulated digestive fractions (5, 2.5, 1.25, and 0.63 mg/mL) were based on previous work by Wagner et al. [40], which tested *Amanita muscaria* extracts on the HMC3 cell line. The concentrations tested by the authors ranged from 50–5000 µg/mL [40]. Exposure of HMC3 cells to different concentrations of simulated digestive fractions resulted in differential effects on cell viability, revealing both species- and dose-dependent responses. In all species, significantly lower cell viability values were registered for 5 mg/mL of simulated digestive fractions, with values ranging between 47.19 and 56.63% viability (Figure 2a–c). At lower concentrations (0.63–2.5 mg/mL), cell viability values were consistently higher than 70%, being considered safe and relevant for biological studies [41].

For the gastrointestinal simulation control (Figure 2d), containing only the INFOGEST enzymes and simulated fluids, cell viability at 2.5 and 5 mg/mL was significantly reduced (15.04 and 25.27%), while lower concentrations maintained viability between 78.22 and 83.30%. These findings suggest that the simulated digestive enzymes and fluids themselves may have detrimental effects on HMC3 cells. Notably, the permeate fractions from TV (Figure 2a), HE (Figure 2b), and PO (Figure 2c) maintained cell viability above 70% at concentrations up to 2.5 mg/mL, indicating better biocompatibility across all species and a protective effect against the digestive enzymes and fluids discussed for the gastrointestinal simulation control.

Regarding gut microbiota metabolites (Figure 3), the concentrations tested were selected based on their physiological relevance and previous literature reporting their effects on neuronal cells. A two-dimensional magnetic resonance spectroscopy study estimated *in vivo* GABA concentrations in the human brain to be approximately 1.01 and 1.16 µmol/cm^3^ for males and females, respectively [42]. Generally, literature data report that *in vivo* GABA concentrations in the brain may range from 1 to 4 mM [43]. The highest concentration tested (50 mM) exhibited significantly reduced cell viability (45.96%), suggesting cytotoxic effects. No significant differences in cell viability were found at lower concentrations tested (3.13–25 mM), with viability values higher than 86.23%. This may suggest that GABA exhibits good biocompatibility with HMC3 cells at physiologically relevant concentrations.

Literature data generally tested concentrations between 10 and 100 μM of SCFAs [44]. All SCFAs were evaluated in concentrations ranging from 6.3 to 100 μM. The results suggest that a concentration of 100 μM of different SCFAs exhibited a cell viability lower than 70%. In the case of acetic acid, cell viability was above 90% across all the other concentrations tested, with no significant differences observed between 6.3 and 50 μM. In contrast, both propionic and butyric acids exhibited concentration-dependent effects on HMC3 cell viability. The cell viability for propionic acid ranged from 70.44% for 50 μM and 95.79% for 6.3 μM, with significant differences between all tested concentrations. For butyric acid, the cell viability ranged from 78.50 and 99.49%, with no significant difference between 12.5 and 25 μM concentrations.

Based on these findings, the concentrations of 0.63–2.5 mg/mL for simulated digestive fractions, 3.13–25 mM for GABA, and 6.3–50 μM for SCFAs were used for subsequent experiments assessing ROS production and antioxidant capacity in HMC3 cells.

#### 3.2.2. Impact of Different Treatments on ROS Production

**Figure 4:**
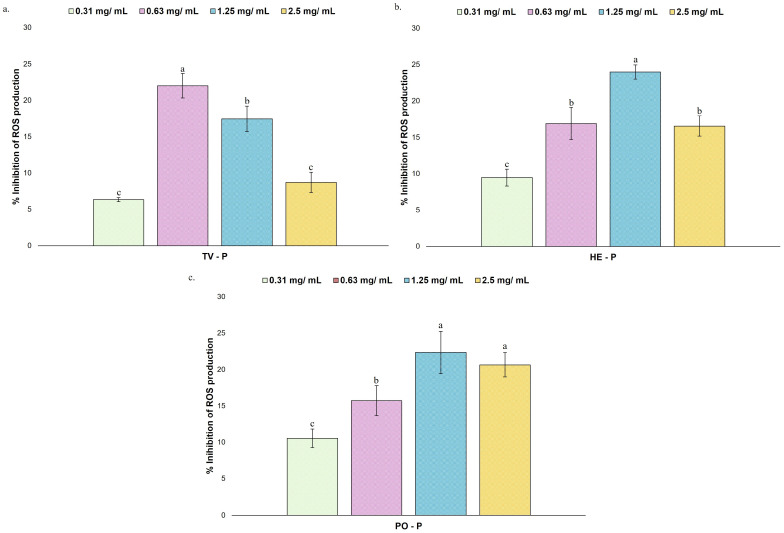
ROS inhibition capacity (%) of HMC3 microglia cells after 24 h exposure to the simulated digestive fractions of TV (**a**), HE (**b**), and PO (**c**) MB at different concentrations (0.63, 1.25, and 2.5 mg/mL). Fluorescence values were normalized using the negative control (basal ROS production), and inhibition was calculated relative to the maximum level of induced ROS (positive control). Bars represent the inhibition of ROS production, as measured by the fluorescent dye DCFH-DA, expressed as a percentage (%) after treatment. In each graph, bars with different superscript letters indicate statistical differences, assessed by one-way ANOVA followed by Tukey’s post hoc test (*p* < 0.05).

**Figure 5:**
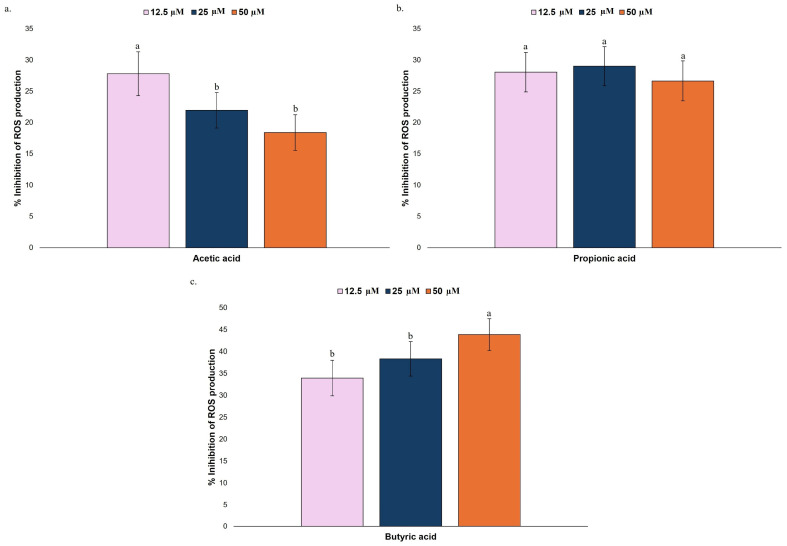
ROS inhibition capacity (%) of HMC3 microglia cells after 24 h exposure to the acetic acid (**a**), propionic acid (**b**), and butyric acid (**c**) at different concentrations (12.5, 25, and 50 µM). Fluorescence values were normalized using the negative control (basal ROS production), and inhibition was calculated relative to the maximum level of induced ROS (positive control). Bars represent the inhibition of ROS production, as measured by the fluorescent dye DCFH-DA, expressed as a percentage (%) after treatment. In each graph, bars with different superscript letters indicate statistical differences, assessed by one-way ANOVA followed by Tukey’s post hoc test (*p* < 0.05).

Oxidative stress leads to the generation of ROS, which plays a key role in the pathogenesis of neurological disorders. The brain, characterized by its high metabolic rate and substantial oxygen consumption, is particularly susceptible to oxidative damage. Excessive ROS accumulation disrupts cellular homeostasis, leading to dysfunction and contributing to neurodegenerative processes. Oxidative stress is involved in several neurological diseases, including AD, PD, and multiple sclerosis [2]. The preventive capacity of simulated digestive fractions and gut microbiota metabolites to inhibit ROS production was assessed. TBHP has been validated as an inducer for oxidative stress and is widely used because it causes lipid peroxidation and impairs mitochondrial function [35]. The production and inhibition of ROS in the tested cell line were further evaluated by using DCFH-DA, an oxidation-sensitive fluorescent probe. The ROS inhibition capacity (%) of the permeate digestive fraction and gut microbiota metabolites is shown in Figure 4 and Figure 5, respectively. Fluorescence values were normalized to the negative control (basal ROS production), and inhibition values were calculated relative to the ROS levels induced in the positive control (maximum level of induced ROS).

Significant inhibition of TBHP-induced ROS production was observed when HMC3 cells were exposed to the permeate fractions of TV, HE, and PO. For TV, the maximum inhibition (22.02%) was observed at 0.63 mg/mL, with a significant decrease at both lower (0.31 mg/mL) and higher concentrations (1.25–2.50 mg/mL). HE showed the highest ROS inhibition (24.00%) at 1.25 mg/mL, while both lower and higher concentrations exhibited significantly reduced ROS inhibitory capacity. For PO, both 1.25 and 2.5 mg/mL resulted in significantly higher ROS inhibition, with values ranging from 20.66 and 22.36%, with statistical differences compared to the lower concentrations. These results suggest a dose-dependent antioxidant effect. However, the concentration at which the most significant inhibition of ROS production occurs varies among species, suggesting that it may be species-specific. Importantly, this variability does not appear to be associated with cytotoxicity, since all samples displayed a similar concentration-dependent effect on viability, with greater toxicity observed at higher concentrations.

The observed ROS inhibition capacities support the neuroprotective potential of mushroom components following gastrointestinal digestion. As far as the authors know, no literature data with mushroom macromolecules or extracts have been published using this specific probe on the HMC3 cell line. Nevertheless, studies using alternative antioxidant assays provide relevant comparisons. Assays with 3,3′,5,5′-tetramethyl-benzidine (TMB) in HMC3 cells have revealed that nanogel systems formulated with *Lentinula edodes* polysaccharides possess ROS-scavenging activity during Aβ-amyloidogenesis [45]. Similar findings were reported for α-glucan extracts from bivalves, which reduced LPS-induced ROS in HMC3 microglia [46]. The dominant bioactive group in the simulated digestive fractions of the current study is α-(1→4)-glucans, which is consistent with their previously reported antioxidant and neuroprotective properties [37,46].

Polysaccharides, together with other identified bioactives in MB such as peptides, unsaturated fatty acids, zinc [37], selenium, GABA, ergosterol, ascorbic acid, carotenoids, tocopherols, and phenolic compounds, likely contribute both through direct ROS scavenging and to indirect modulation of cellular redox signaling pathways. Moreover, since ROS production is mechanistically linked to NF-κB pathway activation in microglia and can trigger inflammatory cascades, the tested fractions may exert broader impacts on neuroinflammation [4].

Future studies should also investigate Nrf2-mediated antioxidant responses, as this pathway is a key player in cellular antioxidant defense [47]. Thus, future studies focusing on the impact of these fractions on the aforementioned pathways may be relevant for fully elucidating the mechanisms behind their action. Complementary assays, including inflammatory and microglial activation markers (e.g., IL-1β, TNF-α, Iba1, CD68), would further provide a more comprehensive characterization of their neuroprotective potential, providing additional strengthening of the mechanistic characterization and helping clarify parallel neuroprotective pathways.

Several studies have also suggested that mushroom macromolecules have a positive impact on modulating intracellular ROS levels in other cell lines under ROS-induced biological systems. *Morchella esculenta* mycelium extract has been shown to reduce endogenous ROS levels in kidney cells in mice with induced acute renal toxicity, in a dose-dependent manner [48]. Additionally, the application of *Flammulina velutipes* polysaccharide extract on H_2_O_2_-stimulated L929 cells (a fibroblast cell line) resulted in a significant decrease in ROS levels [49]. Apparoo et al. [50] reported that ergothioneine extract (a common antioxidant amino acid present in mushrooms) demonstrated ROS scavenging capacity against senescent HT22 neuronal cells. Shao et al. [51] demonstrated that hispidin isolated from *Phellinus* and *Inonotus* species can significantly decrease ROS levels in LPS-stimulated RAW 264.7 macrophages.

Additional *in vivo* studies demonstrated the antioxidant activity of mushroom-derived molecules. For example, supplementation with polysaccharides from *Ganoderma atrum* showed positive potential in oxidative stress and inflammation reduction. An increase in superoxide dismutase, catalase, and glutathione peroxidase activities was reported in rat models with induced inflammation and oxidative damage [52]. Chao et al. [53] also demonstrated that supplementation with an immunomodulatory protein isolated from *Ganoderma microsporum* promotes an increase in superoxide dismutase 1. The findings suggest protective effects against oxidative damage and cognitive impairments after traumatic brain injury [53].

On the other hand, exposure to different concentrations of GABA (3.13–25 mM) did not result in significant ROS inhibition, indicating that this metabolite may not directly contribute to antioxidant defense under the tested conditions. Previous work by Wagner et al. [39] reports that although GABA promotes an increase in the production of IL-8, influencing this inflammatory pathway, this neurotransmitter did not affect ROS production or metabolic rate, corroborating the lack of effect observed here. This suggests that GABA’s neuroprotective effects likely involve alternative mechanisms that are not related to direct oxidative stress modulation.

All tested SCFAs promoted a decrease in intracellular ROS production in HMC3 cells (Figure 5), with distinct dose-dependent profiles. For butyric acid, within the tested concentrations, an increase in concentration resulted in improved inhibition of ROS production (Figure 5c), while for acetic acid, the opposite trend was observed (Figure 5a). A significantly higher inhibition of ROS production was registered with the lower concentration tested (12.5 µM) for acetic acid (27.81%), while for butyric acid, a significant improvement was registered with the higher concentration tested (50 µM), with inhibition of ROS production of 43.88%. No significant differences were found in the concentrations tested of propionic acid, with values ranging from 26.66 to 29.01% of ROS inhibition production (Figure 5b). Similarly to what was observed for the permeate fractions, these results suggest a dose-dependent antioxidant effect for the relevant SCFA tested. As observed for the permeate fractions, these results suggest compound-specific antioxidant effects rather than cytotoxicity-driven responses, as all samples displayed a consistent concentration-dependent effect.

These findings are corroborated by *in vitro* studies suggesting the antioxidant properties of gut microbiota metabolites. Although the neuroprotective effects of propionic acid are reported as limited and indirect [54], a bioinformatics functional study evaluated the possible roles of propionic acid in AD, studying propionic acid-affected genes. This study suggests potential impacts of propionic acid on redox signaling and neuroinflammation [55]. Lactobacilli-derived cell-free supernatants were tested in microglial cells, pointing to protective effects against inflammation and oxidative stress in the brain, through a positive modulation of the Nrf2 pathway [56]. Although the authors do not directly correlate the effects on oxidative stress to SCFAs, lactobacilli are known SCFA producers, and the findings of this study may suggest that these metabolites may play a role in reducing inflammation and potentially modulating oxidative stress in microglial cells. Ahmed et al. [57] also tested numerous cell-free supernatants from gut-derived strains, observing that metabolites produced by *Parabacteroides distasonis* species possess a protective role against ROS in HMC3 and glioblastoma U373 cell lines.

Altogether, the findings obtained herein suggest that both simulated digestive fractions and SCFAs may modulate oxidative stress in microglia. As previously mentioned, future studies should include a detailed investigation of inflammatory pathways, microglial activation markers, and key antioxidant signaling cascades (e.g., Nrf2, NF-κB) to fully elucidate the mechanisms underlying these protective effects.

### 3.3. Caenorhabditis Elegans-Based Assays

The CL2355 strain expresses the Aβ peptide in neurons, leading to defects in chemotactic behavior. Pan-neuronal expression of the Aβ_1–42_ gene in this strain requires induction by increased temperature [23,28]. The CL2122 strain, with a wild-type phenotype, was used as a control worm for CL2355 [23]. The BR5270 strain pan-neuronally expresses a gene encoding a pro-aggregant fragment of human Tau (htau) protein with the deletion of the amino acid K280 (htauK280). This strain exhibits impaired motility and neuronal dysfunction [24,27,28,58]. The CL4176 strain was used for paralysis assays. This strain expresses the human Aβ_1–42_ gene in the body wall muscle cells, making it suitable for investigating the potential protective effects of MB against Aβ-induced toxicity *in vivo*. When transgene expression is induced by a temperature increase, the CL4176 strain exhibits a paralysis phenotype, which can be easily monitored over time [23,28].

#### 3.3.1. Impact of Different Treatments and Concentrations on Neuronal Tau Expression-Induced Defects in Chemotaxis Behavior

Neurodegeneration, often linked to the presence of pathological forms of Tau within the nervous system, is a hallmark of tauopathies, with progressive memory impairment being the most common symptom observed in patients [27]. An initial screening was conducted to evaluate the effects of different concentrations of gut-microbiota metabolites (SCFAs and GABA) and permeate fractions resulting from gastrointestinal digestion and 3.5 kDa dialysis (simulated digestive fractions) of TV, HE, and PO on chemotaxis assays using the BR5270 strain.

##### Gut Microbiota Metabolites

As previously mentioned, *in vitro* human fecal fermentation results suggested that consuming these MBs considerably increases the production of gut microbiota metabolites, including acetic, propionic, and butyric acids, as well as GABA. Zheng et al. [26] tested cecal samples from mice fed resistant starch in wild-type *C. elegans* (N2 strain). The samples resulting from fermentation were highly complex, containing residual fecal inoculum and a diverse range of metabolic byproducts. The authors report that using individual SCFAs provides more detailed insights into their impact on intestinal fat deposition. Indeed, the use of fermentation products complicates studies and the evaluation of gut metabolites effects in *C. elegans* models. Thus, the present study tested individual standards of the primary gut metabolites to better understand their specific effects.

**Figure 6:**
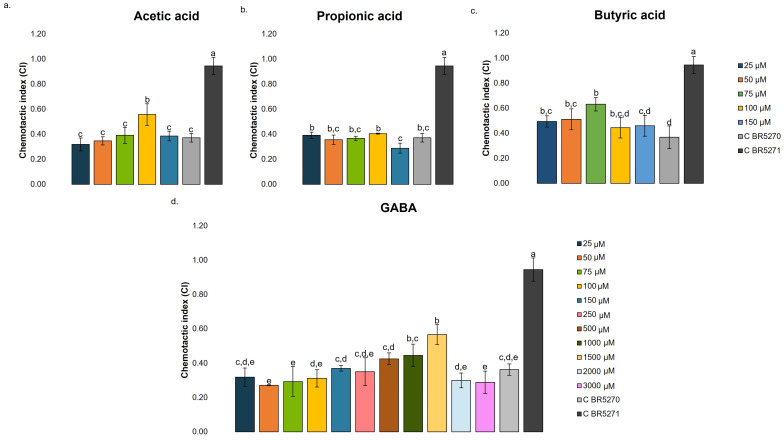
Chemotaxis behavior in strain BR5270 treated with different concentrations of short-chain fatty acids (SCFA; 25, 50, 75, 100, and 150 µM), GABA (25, 50, 75, 100, 150, 250, 500, 1000, 1500, and 3000 µM), not treated (0.1% DMSO; C BR5270), and in BR5271 (0.1% DMSO; C BR5271) that expresses anti-aggregating Tau protein: (**a**) acetic acid; (**b**) propionic acid; (**c**) butyric acid, and (**d**) GABA. All determinations were carried out in triplicate (250 worms per plate), and the result was expressed as mean ± standard deviation. In each graph, bars with different superscript letters indicate statistical differences, assessed by one-way ANOVA followed by Tukey’s post hoc test (*p* < 0.05).

The CI of the BR5270 strain treated with different SCFAs concentrations and without treatment (0.1% DMSO; C BR5270), as well as in the BR5271 control strain (0.1% DMSO; C BR5271), is shown in Figure 6. The untreated BR5270 strain exhibited a significantly reduced CI value (0.37) compared to the control BR5271 strain (0.95). This corroborated the severe cognitive defects present in the BR5270 strain and aligned with recent reports of the CI of this strain grown under similar conditions [24].

The starting SCFAs concentrations tested were based on literature data that generally focus on concentrations between 10 and 100 μM of SCFAs [44], and in the cell viability assays in the HMC3 cell line presented in the previous section. Accordingly, several concentrations closer to those values, namely, 25, 50, 75, 100, and 150 µM, were assayed. The treatment with acetic acid improved the chemosensory response of the BR5270, particularly at a concentration of 100 µM (0.56), with a significantly higher CI value (Figure 6a). Similarly, treatment with butyric acid (Figure 6c) showed a significant improvement in cognitive defects at 25, 50, and 75 µM, with the highest CI value observed at 75 µM (0.63). Both metabolites displayed a clear dose-dependent pattern within this concentration range. In contrast, treatment with propionic acid (Figure 6b) did not significantly improve chemotaxis behavior across the tested concentrations.

The CI values of different treatments must be interpreted in relation to controls, rather than being taken as absolute values. Accordingly, treatment with acetic acid (100 µM) and butyric acid (75 µM) increased chemotaxis behavior in approximately 51 and 70% respectively, compared with BR5270 without treatment. These findings suggest that acetic (100 µM) and butyric acid (75 µM) may provide neuroprotective effects *in vivo*, helping to reverse cognitive defects caused by Tau aggregation. Concerning propionic acid, it is more strongly associated with metabolic and immunomodulatory roles, while its direct neuroprotective effects remain limited [54].

Walker et al. [59] evaluated the impact of *C. elegans* gut colonization with pathogenic bacteria and butyrogenic bacteria on host proteostasis. Although different strains were used, the authors demonstrated that enteric pathogens promote polyglutamine aggregation and proteotoxicity, while commensal butyrogenic bacteria suppress these effects. These findings further support the protective role of butyrate against proteotoxic stress, consistent with the results obtained in the present study.

As previously discussed, literature data suggest that *in vivo* GABA concentrations in the brain may range from 1 to 4 mM [43]. The observations made in the HMC3 cell line indicated that GABA concentrations of 50 mM adversely affected cell viability (see Figure 3a). Accordingly, several concentrations were tested close to this range to determine the concentration associated with the highest CI value and the threshold where CI starts to decrease. The CI of the BR5270 strain treated with the neurotransmitter GABA at different concentrations (25, 50, 75, 100, 150, 250, 500, 1000, 1500, and 3000 µM) is also shown in Figure 6d. GABA significantly improved CI at 1000 and 1500 µM, with the highest CI observed at 1500 µM (0.57). At this concentration, CI increased by approximately 54% relative to the untreated BR5270 control, and a dose-dependent effect was observed.

GABA has been suggested as one of the most effective gut-derived metabolites influencing the central nervous system [60]. Urrutia et al. [60] evaluated the effect of various dietary GABA-producing bacteria supplemented with GABA, glutamic acid, and lactate, each at 2 mM, on a wild-type *C. elegans* (N2) and neurodegeneration mutant strains (TU2773, CF1139, WCH34, WCH39, WCH40, TU38, TU3755, WCH6, and WCH41). Specifically, TU2773 is a strain that expresses the human Tau protein. The results suggested that GABA produced by the tested bacteria and other metabolites, such as glutamic acid (a precursor of GABA) and lactate, exerted a neuroprotective effect [60]. These findings support the present results, reinforcing that GABA may modulate Tau-induced dysfunction and contribute to neuronal homeostasis.

##### Simulated Digestive Fraction of Mushroom Biomasses

**Figure 7:**
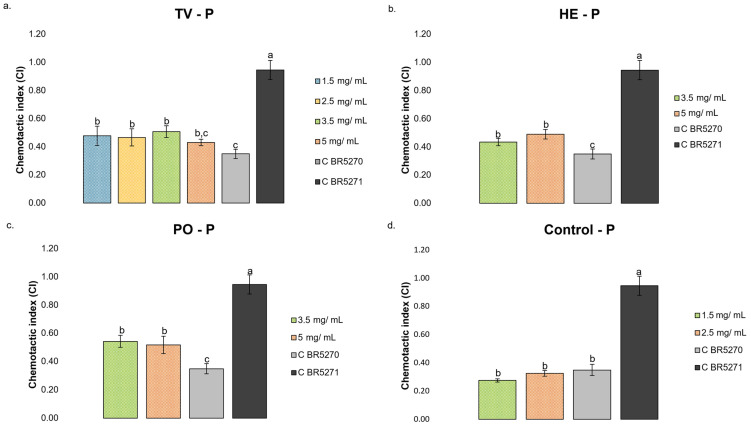
Chemotaxis behavior in strain BR5270 treated with different concentrations of simulated digestive fractions (1.5, 2.5, 3.5, and 5 mg/mL) of *Trametes versicolor* (TV), *Hericium erinaceus* (HE), *Pleurotus ostreatus* (PO), and gastrointestinal control, and not treated (0.1% water; C BR5270), and in BR5271 (0.1% water; C BR5271) that expresses anti-aggregating Tau protein: (**a**) TV—P; (**b**) HE—P; (**c**) PO—P; (**d**) Control—P. All determinations were carried out in triplicate (250 worms by plate), and the result was expressed as mean ± standard deviation. In each graph, bars with different superscript letters indicate statistical differences, assessed by one-way ANOVA followed by Tukey’s post hoc test (*p* < 0.05).

The CI in the presence of simulated digestive fractions of different MB species is shown in Figure 7.

The simulated digestive fractions derived from TV species demonstrated a significantly higher CI at 3.5, 2.5, and 1.5 mg/mL, with the highest CI observed at 3.5 mg/mL (Figure 7a). As the three species fractions possessed similar chemical composition, only 5 and 3.5 mg/mL concentrations were tested for HE (Figure 7b) and PO (Figure 7c), observing improved CI at both concentrations. The highest CI was found at 5 mg/mL for HE and 3.5 mg/mL for PO, although no significant differences existed between the two tested concentrations in any of the species. The treatments with TV (5 mg/mL), HE (3.5 mg/mL), and PO (5 mg/mL) simulated digestive fractions resulted in an increase in chemotaxis behavior in approximately 38, 32, and 46% compared with control BR5270 without treatment, respectively.

As the tested fractions result from gastrointestinal digestion, they include compounds derived from MB as well as enzymes, fluids, and bile salts, potentially impacting the chemotactic behavior of *C. elegans* strains. Accordingly, the control sample (blank) of gastrointestinal fluids, enzymes, and bile salts used during the INFOGEST protocol was also tested. According to the chemical characterization, in the simulated digestive fractions of TV, HE, and PO, less than 50% of the composition corresponded to gastrointestinal fluids, enzymes, and bile salts. In this context, approximate concentrations of 1.5 and 2.5 mg/mL were tested for the control sample, without observing a significant effect (Figure 7d), which suggested that the observed reversal effect of Tau damage may result from bioactive compounds resulting from MB digestion.

To the best of our knowledge, this is the first study to test the neuroprotective effect of compounds resulting from MB digestion, providing new insights into their bioactive potential and highlighting the novelty of this approach.

#### 3.3.2. Impact of Gut Microbiota Metabolites and Simulated Digestive Fractions on Neuronal Aβ Expression-Induced Defects in Chemotaxis Behavior

**Figure 8:**
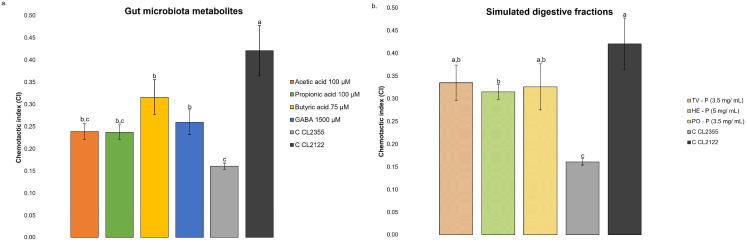
Chemotaxis behavior in strain CL2355 treated with (**a**) different gut microbiota metabolites (acetic acid: 100 µM; propionic acid: 100 µM; butyric acid: 75 µM; GABA: 1500 µM and Mix: acetic acid 100 µM, butyric acid: 75 µM and GABA: 1500 µM), (**b**) simulated digestive fraction of *Trametes versicolor* (TV; 3.5 mg/mL), *Hericium erinaceus* (HE; 5 mg/mL), and *Pleurotus ostreatus* (PO; 3.5 mg/mL) and not treated (0.1% DMSO; C CL2355) and in CL2122 (0.1% DMSO; C CL2122), which presents a wild-type phenotype under the same conditions. All determinations were carried out in triplicate (250 worms by plate), and the result was expressed in mean ± standard deviation. In each graph, bars with different superscript letters indicate statistical differences, assessed by one-way ANOVA followed by Tukey’s post hoc test (*p* < 0.05).

The accumulation of toxic plaques with insoluble Aβ is believed to be the main event that triggers neuronal degeneration in AD [4]. Therefore, the chemotaxis behavior of age-synchronized worms of the CL2355 strain cultured with and without treatments was assessed and compared with the CL2122 strain (control). This assay evaluates the ability of *C. elegans* strains to move in response to a chemical odorant (attractant sites with benzaldehyde vs. control sites), which is related to the capacity of the metabolites under study to improve the behavior of worms that express Aβ peptide in neurons after induction by temperature. For the study, the concentrations of gut microbiota metabolites (acetic acid: 100 µM; propionic acid: 100 µM; butyric acid: 75 µM; and GABA: 1500 µM) and simulated digestive fractions (TV and PO: 3.5 mg/mL; and HE: 5 mg/mL) that exhibited higher CI values in the BR5270 strain were selected. The results are presented in Figure 8. As shown, the untreated CL2355 strain exhibited a significantly lower CI value (0.16) than the control CL2122 strain (0.42). These observations align with literature data describing assays performed under similar conditions [23].

Treatments with butyric acid and GABA (Figure 8a) contributed to normalizing the chemosensory response in CL2355, significantly increasing CI compared to the untreated CL2355 control. Compared with the CI of control CL2355 without treatment under the same conditions, butyric and GABA resulted in an improvement in 100 and 62.5% of CI. Although other treatments exhibited higher CI compared to the untreated CL2355, no statistical differences were found. These results suggest that the treatment with 75 µM of butyric acid and 1500 µM of GABA mitigates amyloid-induced neuronal toxicity.

No data on the direct effect of these metabolites on CL2355 chemotaxis behavior was found in the literature. Although not targeting the same strains used in the present work, previous research has demonstrated the positive impact of GABA and butyric acid on proteotoxicity [59,60]. For instance, Urrutia et al. [60] evaluated the effects of GABA and other metabolites on various neuroprotective mutant strains, including WCH34, WCH39, and WCH40 that express Aβ peptides, mimicking AD-related neurotoxicity. The findings from the present study and previous reports highlight the high potential of GABA and butyric acid to counteract neurotoxicity.

Chemotaxis testing on the CL2355 strain was also performed with the simulated digestive fractions of TV, HE, and PO, and the results are presented in Figure 8b. The CI of the CL2355 strain treated with simulated digestive fractions of the three species was significantly higher than that of the untreated strain, in the case of TV and PO, without significant differences with that of the CL2122 control strain. These findings support the potential use of bioactive compounds from MB digestion in the management of neurodegenerative diseases, suggesting possible *in vivo* protection against amyloid neurotoxicity.

Kittimongkolsuk et al. [25] tested extracts of *Lignosus rhinoceros* mushroom in CL2355 and AM141 (a strain exhibiting PolyQ40 aggregation). It was found that treatment with ethanolic extracts (50, 100, and 200 µg/mL) significantly improved the CI in both *C. elegans* strains, while considerably decreasing PolyQ40 aggregation. Zhang et al. [61] also tested the ethanolic extract of *Dictyophora indusiate* (1 mg/mL) in CL2355 and other transgenic strains, reporting a significant increase in CI. The results suggested that both Aβ-mediated and polyQ dysfunctions were alleviated. Additionally, ergosterol, an important bioactive compound found in mushrooms, was reported to enhance chemotaxis behavior in the CL2355 strain. The results suggested that ergosterol, at concentrations of 25 and 50 nM, inhibits Aβ synthesis and enhances longevity in Aβ-overexpressing *C. elegans* strains [62]. The effects found in the present study, where CL2355 was treated with the digestive fraction of MB, would be in line with those observed in those reports.

#### 3.3.3. Impact of Gut Microbiota Metabolites and Simulated Digestive Fractions on Body Paralysis in the CL4176 Strain

In the paralysis assay, the Aβ_1–42_ gene was induced in the body wall muscle cells of CL4176 worms by raising the temperature from 16 to 25 °C, resulting in muscle paralysis. The number of paralyzed worms was monitored to assess Aβ-induced paralysis and progression after 30 h of temperature upshift. Previous studies under the same conditions indicate no significant differences in paralysis between 24 and 28 h, with most worms remaining motile in all groups, including the control [23]. Therefore, the percentage of non-paralyzed worms was monitored from 30 to 34 h, which corresponds to the time window where amyloid-induced paralysis becomes evident and biologically relevant.

**Figure 9:**
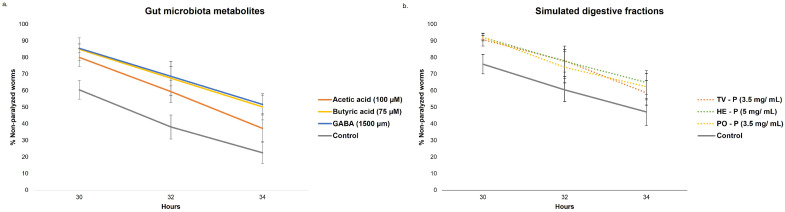
Progression of paralysis in transgenic *C. elegans* CL4176 strain treated with (**a**) gut microbiota metabolites (acetic acid: 100 µM; butyric acid: 75 µM; GABA: 1500 µM), (**b**) simulated digestive fraction of *Trametes versicolor* (TV—P; 3.5 mg/mL), *Hericium erinaceus* (HE—P; 5 mg/mL), and *Pleurotus ostreatus* (PO—P; 3.5 mg/mL), and an untreated control (0.1% DMSO). Expression of the β-amyloid peptide was induced by shifting the temperature to 25 °C after 48 h at 16 °C. Worms were scored for paralysis every 2 h from 30 to 34 h post-induction. Data represent the mean of three independent experiments (*n* = 100 per group).

Figure 9a shows the progression of non-paralyzed worms treated with different SCFAs and GABA, as well as an untreated control (0.1% DMSO; control). All treatments resulted in a higher percentage of non-paralyzed worms between 30 and 34 h than the control group (60.5, 38.1, and 22.5% at 30, 32, and 34 h, respectively). A significant delay in paralysis was observed with butyric acid (75 µM), acetic acid (100 µM), and GABA (1500 µM) treatments (log-rank test, *p* < 0.001).

The progression of non-paralyzed worms treated with different simulated digestive fractions, as well as an untreated control (0.1% water; control), is presented in Figure 9b. The treatment with the three species resulted in a higher percentage of non-paralyzed worms between 30 and 34 h than the control group (76.0, 60.5, and 47.2% at 30, 32, and 34 h, respectively). A significant delay in paralysis was observed with TV (3.5 mg/mL), HE (5 mg/mL), and PO (3.5 mg/mL) treatments (log-rank test, *p* < 0.05). These findings suggested that gut microbiota metabolites (acetic acid, butyric acid, and GABA) and simulated digestive fractions of MB can alleviate Aβ toxicity in muscles.

The paralysis assay results revealed that both MB simulated digestive fractions and gut microbiota metabolites—particularly acetic acid, butyric acid, and GABA—significantly delayed the onset of amyloid-induced paralysis in the CL4176 strain. Further detailed mechanistic studies are needed to expand this knowledge. To the best of the authors’ knowledge, no previous studies have directly tested mushroom macromolecules or gut microbiota-derived metabolites in the CL4176 paralysis assay, highlighting the novelty of this work. However, different studies have suggested the potential of bioactive compounds, such as flavonoids including epicatechin and quercetin [23,24], in modulating paralysis onset. Additionally, Shi et al. [63] evaluated the effect of monascin, a secondary metabolite derived from an edible fungus, on oxidative stress and Aβ accumulation in *C. elegans* strains, including paralysis delay in CL4176. This metabolite significantly reduced ROS production and delayed paralysis onset.

### 3.4. Result Integration

All MB species tested—TV, HE, and PO—significantly decreased ROS production in the HMC3 cell line, suggesting that bioactive compounds resulting from digestion can modulate oxidative stress responses relevant to AD. Optimal inhibitory effects were species-dependent, with the most effective concentrations ranging from 0.63 to 2.5 mg/mL, and ROS inhibition between 20 and 24%. These values align with antioxidant effects reported for other mushroom-derived polysaccharides or bioactive macromolecules present in MB in microglial or neuronal cell lines [45,46,48,49,50,51]. GABA did not result in significant ROS inhibition, indicating that this metabolite may not exert a direct antioxidant effect in microglia under the tested concentrations and oxidative stress conditions induced by TBHP. Among the SCFAs, butyric acid (50 µM) showed the most pronounced ROS inhibition (44%), followed by propionic acid (12.5–50 µM) and acetic acid (12.5 µM). These findings demonstrate that both MB simulated digestive fractions and specific SCFAs exhibit significant antioxidant activity in microglial cells.

The study also highlights the significant role of gut microbiota-derived metabolites in mitigating Tau aggregation and neurotoxicity, with a dose-dependent effect observed. Acetic acid (100 µM), butyric acid (75 µM), and GABA (1500 µM) significantly improved chemosensory responses in the BR5270 strain. Although propionic acid did not significantly improve chemotaxis, this limited efficacy is consistent with previous reports suggesting that propionic acid may exert more indirect neuroprotective effects, potentially through modulation of inflammatory pathways rather than directly acting on neuronal function [54]. The results also suggested that butyric acid and GABA treatments effectively restored chemosensory responses in the CL2355 strain, indicating a protective effect against amyloid-induced neuronal dysfunction. Furthermore, both metabolites significantly delayed the onset of paralysis in the Cl4176 strain. These findings support the *in vivo* protective effects of butyric acid and GABA against hyperphosphorylated Tau neurotoxicity and amyloid toxicity in neurons and muscles, likely mediated via the gut-brain axis.

For the simulated digestive fractions, all mushroom species significantly improved chemosensory responses in the BR5270 and CL2355 strains, with the most significant effects observed for TV and PO at 3.5 mg/mL and for HE at 5 mg/mL. All species also significantly delayed paralysis progression in the CL4176 strain, reinforcing their neuroprotective potential. These results provide valuable insights into the beneficial effects of MB compounds derived from the digestion against Aβ- and Tau-induced toxicity.

The antioxidant and neuroprotective effects observed for simulated digestive fractions of TV and HE are in line with previous studies that report an improvement in hippocampal function, activation of defense mechanisms relevant to AD models [12,13,17,29]. The present work extends these findings, suggesting that digestive fractions (representing potentially absorbable metabolites) and gut-derived metabolites can contribute to neuroprotection.

In addition, the present work also demonstrated the neuroprotective potential of PO species, demonstrating that digested biomass fractions can modulate chemotaxis behavior and delay paralysis. The differences among TV, HE, and PO simulated digestive fractions in ROS inhibitory capacity and in chemotaxis and paralysis behavior reflect species-specific compositional profiles, including variations in α- and β-glucans, peptide profile, zinc [37], ergosterol, phenolic compounds, and other antioxidant macromolecules.

## 4. Conclusions

This study demonstrates that simulated digestive fractions of MBs and key gut microbiota metabolites, particularly SCFAs and GABA, exert significant antioxidant and neuroprotective effects in both *in vitro* (HMC3 microglial cells) and *in vivo* (*C. elegans*) models of neurodegeneration. Together, these findings support the relevance of mushroom-derived compounds and micrsobiota-related metabolites as contributors to the attenuation of ROS, Aβ- and Tau-induced toxicity, reinforcing the potential of an MB-based diet as a preventive nutritional strategy against neurodegenerative disorders.

Future studies should explore the blood–brain barrier permeability of these compounds, integrate metabolomic and transcriptomic analyses to identify molecular targets, and evaluate synergistic effects between mushroom fractions and gut microbiota metabolites. In addition, including inflammatory and microglial activation markers (e.g., IL-1β, TNF-α, Iba1, CD68) and key antioxidant pathways such as Nrf2 would further strengthen the mechanistic understanding of the observed effects. Such approaches will help advance their translational potential for preventive and therapeutic applications in human neurodegenerative diseases.

## Data Availability

The original contributions presented in this study are included in the article. Further inquiries can be directed to the corresponding author.
